# *Akkermansia muciniphila*: A Potential Target for the Prevention of Diabetes

**DOI:** 10.3390/foods14010023

**Published:** 2024-12-25

**Authors:** Kairu He, Feiyu An, Henan Zhang, Danli Yan, Tong Li, Junrui Wu, Rina Wu

**Affiliations:** 1 College of Food Science, Shenyang Agricultural University, Shenyang 110866, China; kairuhe0705@163.com (K.H.);; 2 Shenyang Key Laboratory of Microbial Fermentation Technology Innovation, Shenyang 110866, China; 3 Liaoning Engineering Research Center of Food Fermentation Technology, Shenyang 110866, China

**Keywords:** *Akkermansia muciniphila*, diabetes, gut microbiota, drug, diet, GLP-1, insulin resistance

## Abstract

*Akkermansia muciniphila*, a Gram-negative anaerobic bacterium colonizing the intestinal mucus layer, is regarded as a promising “next-generation probiotic”. There is mounting evidence that diabetes and its complications are associated with disorders of *A. muciniphila* abundance. Thus, *A. muciniphil* and its components, including the outer membrane protein Amuc_1100, *A. muciniphila*-derived extracellular vesicles (AmEVs), and the secreted proteins P9 and Amuc_1409, are systematically summarized with respect to mechanisms of action in diabetes mellitus. Diabetes treatments that rely on altering changes in *A. muciniphila* abundance are also reviewed, including the identification of *A. muciniphila* active ingredients, and dietary and pharmacological interventions for *A. mucinihila* abundance. The potential and challenges of using *A. muciniphila* are also highlighted, and it is anticipated that this work will serve as a reference for more in-depth studies on *A. muciniphila* and diabetes development, as well as the creation of new therapeutic targets by colleagues domestically and internationally.

## 1. Introduction

Diabetes mellitus is a complex chronic metabolic disease characterized by complications such as kidney failure, heart attack, and stroke. According to the World Health Organization (WHO), diabetes ranked as the ninth most prevalent cause of death globally in 2019 [1], directly accounting for 1.5 million deaths. More than 420 million people worldwide had diabetes in 2021, and this figure is anticipated to surge to 578 million by 2030. It is of utmost importance to decipher the pathophysiology of diabetes and formulate effective treatment modalities. By preventing or detecting diabetes at an early stage and increasing treatment options for all forms of diabetes, especially in middle-income countries, a large number of premature deaths could be avoided. Substantial research efforts are being devoted to exploring the different etiologies that may trigger diabetes, including gut flora, blood metabolites, and other factors. Recent evidence highlights the link between gut flora and diabetes progression. Changes in microbiota composition have been observed in both type 1 diabetes (T1D), type 2 diabetes (T2D), and gestational diabetes mellitus (GDM) patients. The origin of the pathological process was suspected to be related to pro-inflammatory effects, increased intestinal permeability, glucose and lipid metabolism, and the development of insulin resistance [2]. As research progresses, the role and mechanisms of intestinal flora in diabetes are being revealed. The intestinal flora has shown some promise in the prevention, early detection, diagnosis, and treatment of diabetes mellitus, according to animal research and a few clinical investigations. Among the different bacteria observed, *Akkermansia muciniphila* (*A. muciniphila*) is a recurrent specific bacterium with considerable potential [3].

*A. muciniphila*, a Gram-negative bacteria isolated from human feces, was identified in 2004 [4]. It mostly colonizes the outer mucus layer of the gastrointestinal tract. It was named after the Dutch microbial ecologist Anton DL Akkermans in honor of his contributions to the field. *A. muciniphila* is a human intestinal commensal bacterium that can use mucins as its sole source of carbon, nitrogen, and energy to provide energy to fuel epithelial cells. It has an essential role in the intestinal barrier, mucus production, and mucus layer thickness [5]. Mucins are an important component of the intestinal epithelial mucus layer. Mucins are highly glycosylated molecules produced by intestinal epithelial cup cells and have a protein backbone decorated with multiple carbohydrate chains. They form a transparent mucus layer on the surface of intestinal tissue and act as the primary habitat for a variety of intestinal microorganisms. To ward against invaders and maintain the integrity of the intestinal barrier, *A. muciniphila* can utilize the glycans and amino acids in the mucin peptide backbone as a source of energy [6]. A reduction in *A.muciniphila* may have an effect on the thickness of the intestinal mucosal layer, weakening the intestine’s ability to act as a barrier against pathogenic bacteria and toxic substances that might cause disease. In people with obesity, diabetes, and inflammatory bowel disease, *A. muciniphila* concentrations are often lower. *A. muciniphila* is closely associated with host health, energy metabolism, and immune response, participating in the regulation of immune, metabolic, neurological, and other physiological processes. Its metabolic characteristics and regulation mechanisms are a current area of intense research. In recent years, with the deepening of microbial structure understanding and the application of multi-omics technologies, significant progress has been made in the research related to *A. muciniphila*. The mechanisms by which *A. muciniphila* exerts its probiotic properties have been gradually revealed with the aid of extensive genome sequencing analysis. Due to their distinctive probiotic properties, *A. muciniphila* is widely regarded as the next generation of probiotics for treating diabetes [7], obesity [8], and other diseases. This review summarizes the regulatory mechanisms of *A. muciniphila* and its components in T1D, T2D, and GDM. Pro-inflammatory effects, altered intestinal permeability and endotoxin levels, the emergence of insulin resistance, and GLP-1 secretion are all linked to these pathways. It is proposed that drugs and diet induce changes in the abundance of *A. muciniphila* and mediate the development of diabetes, and that *A. muciniphila* may grant potential targets for the treatment of diabetes.

## 2. Mechanism of Action of *A. muciniphila* in Diabetes Mellitus

### 2.1. Role of A. muciniphila in T1D

**Figure 1:**
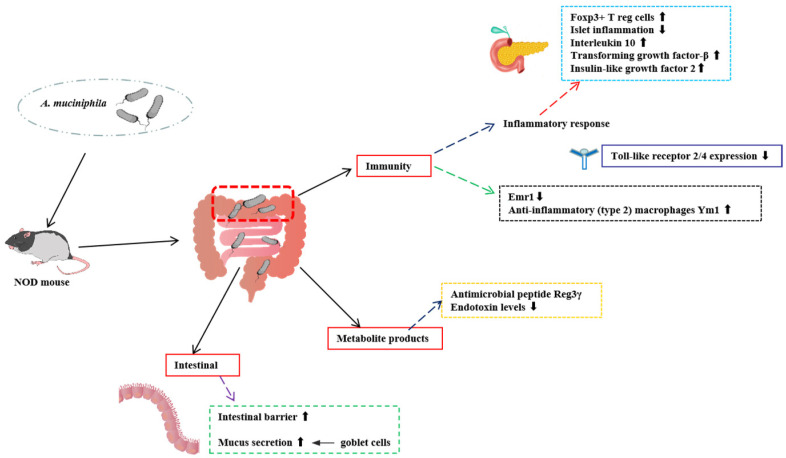
Mechanisms of *A. muciniphila* regulation of homeostasis in patients with T1D. Mechanistically, the administration of *A. muciniphila* enhanced the intestinal barrier, stimulated mucus secretion by goblet cells, and maintained intestinal homeostasis. While decreasing the expression of Emr1, a marker of inflammatory and other macrophages, *A. muciniphila* increased the expression of the anti-inflammatory (type 2) macrophage Ym1. *A. muciniphila* increased the expression levels of metabolites such as the antimicrobial peptide Reg3γ, thereby promoting the separation between the microbiota and gut epithelium, and reduced serum levels of endotoxin. *A. muciniphila* treatment attenuated islet inflammatory effects, which reduced TLR expression. It also increased the number of regulatory Foxp3+ T reg cells and promoted the expression of IL-10 and TGF-β, substances with anti-inflammatory properties. Simultaneous heat inactivation of *A. muciniphila* increased metabolic signaling pathways in the gut and promoted the secretion of IGF2 to regulate T1D. NOD: non-obese diabetic; TLR: Toll-like receptor; IL-10: interleukin 10; TGF-β: transforming growth factor-β; IGF2: insulin-like growth factor 2. ⬆ indicates an increase in the level of substance expression and ⬇ indicates a decrease in the level of substance expression.

T1D, also known as insulin-dependent diabetes mellitus, is an autoimmune disease characterized by defective insulin secretion by pancreatic β-cells [9]. Current knowledge of its pathophysiology and prevention measures is limited. Individuals with T1D present a less stable and diverse gut microbiota. It has been discovered that the abundance of *A. muciniphila* is genetically correlated with an elevated risk of autoantibodies associated with T1D [10]. *A. muciniphila* may be a potential probiotic for the treatment of T1D (Figure 1).

Non-obese diabetic (NOD) mice are widely used as an animal model for T1D [11]. Early experiments showed that *A. muciniphila* was the predominant gastrointestinal flora in NOD mice treated orally with the antibiotic vancomycin in early childhood and was accompanied by a lower incidence of T1D, suggesting that *A. muciniphila* may be a key flora in delaying T1D [12]. Other research has backed up the *A. muciniphila* effect based on the NOD mouse model. Hänninen A compared and analyzed the colonization of NOD mice with high and low incidences of diabetes, and found that *A. muciniphila* was absent in the group with higher prevalence in the T1D mouse model [10]. A study by Fassatoui et al. [13], which revealed that patients with T1D had lower proportions of *A. muciniphila* and *Faecalibacterium prausnitzii* than persons without diabetes, further corroborated this conclusion. Low concentrations of *A. muciniphila* in the intestine may indicate a thinner mucus layer, which compromises the function of the intestinal barrier and increases the translocation of bacterial toxins [13]. *A. muciniphila* was implanted into high-prevalence NOD mice by Hänninen et al. using tube feeding [10]. The results showed that the transfer of *A. muciniphila* enhanced the intestinal barrier, stimulated mucus secretion, and significantly postponed the development of diabetes. A recent study showed that streptozotocin (STZ)-injected mice exhibited increased *A. muciniphila* abundance, and that gavage of heat-killed *A. muciniphila* protected mice from STZ-induced hyperglycemia and atrophy by promoting intestinal insulin-like growth factor 2 (IGF2) secretion [14]. The modulation of T1D by *A. muciniphila* was seen to correlate with an increase in the expression of the antimicrobial peptide Reg3γ, an increase in the anti-inflammatory (type 2) macrophage Ym1, and a decrease in endotoxin [10,11,12,13]. Delayed T1D by *A. muciniphila* was accompanied by an augmentation of the immune response, including a decrease in the expression of Toll-like receptors (TLRs) in the pancreatic islets, an increase in the number of Foxp3+ T reg cells, and elevated levels of IL-10 and TGF-β in pancreatic draining lymph nodes. TLR has been shown to play an important role in the immunopathogenesis of T1D in animal models and in humans by inducing innate immune responses and developing adaptive immunity through sensing of specific ligands of exogenous microorganisms [15,16]. Elevated IL-10 and TGF-β levels in pancreatic draining lymph nodes have been suggested to be a response to colonic innate immune signaling [17]. In conclusion, these studies elucidate the role of *A. muciniphila* in T1D, and live or heat-killed *A. muciniphila* may be a potential therapeutic target for T1D.

### 2.2. Role of A. muciniphila in T2D

**Figure 2:**
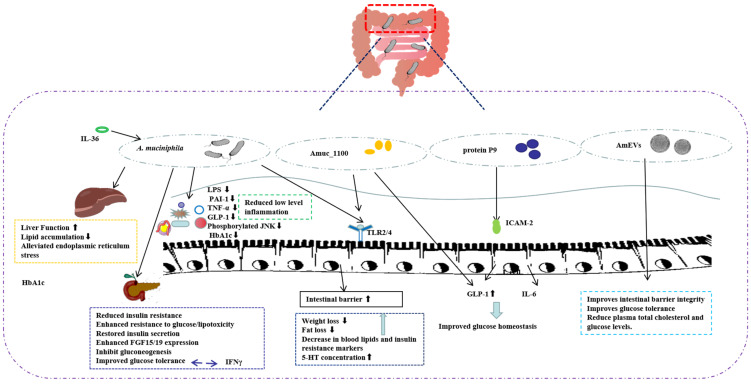
Proposed mechanisms of *A. muciniphila* in regulating glucose homeostasis in T2D. It has been demonstrated that *A. muciniphila*, along with its active components Amuc 1100, P9 protein, and AmEVs, controls glucose homeostasis and mitigates the progression of T2D. Mechanistically, the oral administration of *A. muciniphilaa* improved liver function, reduced lipid accumulation, and alleviated endoplasmic reticulum stress in type 2 diabetic rats. By decreasing inflammatory markers like TNF-α, LPS, and PAI-1, *A. muciniphila* supplementation attenuated low-level inflammation. Live *A. muciniphila* also restored insulin secretion and enhanced FGF15/19 expression to inhibit gluconeogenesis. Additionally, *A. muciniphila* modulated the inflammatory cytokine IFNγ to shield mice from the harmful effects of glucose intolerance. Amuc_1100 acted on TLR2/4, strengthened the intestinal barrier, and reduced blood lipids and insulin resistance markers. To improve glucose homeostasis, the secreted protein P9 can attach to the ligand ICAM-2 and cause L cells to release GLP-1. These results were also found to apply to Amuc 1100. AmEVs can enhance intestinal tight junctions and improve glucose tolerance. AmEVs: *A. muciniphila*-derived extracellular vesicles; LPS: lipopolysaccharide; PAI-1: plasminogen activator inhibitor-1; FGF15/19: fibroblast growth factor 15/19. ⬆ indicates an increase in the level of substance expression and ⬇ indicates a decrease in the level of substance expression.

T2D is a common metabolic disorder that accounts for more than 90% of diabetic patients and is critically characterized by hyperglycemia, decreased insulin secretion, and insulin resistance [18]. The prevalence of T2D has shown a global trend of annual increase. In addition to the recognized genetic and environmental risk factors, the modification of the gut flora has some potential for the treatment and prevention of T2D [19,20]. As shown in Figure 2, the *A. muciniphila* and its components have been studied by various researchers to reduce the risk of developing T2D.

#### 2.2.1. *A. muciniphila* and T2D

In recent years, there have been numerous studies on microorganisms in patients with T2D. An early macrogenomic association study showed that some genes belonging to *A. muciniphila* were enriched in T2D patients [21]. Additionally, another study also showed a reduced abundance of *A. muciniphila* in obese and T2D mice [22]. The most significant characteristics of the gut microbiota of people with type 2 prediabetes are the reduced abundance of *Clostridium* and *A. muciniphila* [23], which is similar to that seen in chronic disorders with low levels of inflammation. Viable *A. muciniphila* treatment can counteract diet-induced metabolic endotoxemia, reduce plasma glucose levels, and improve glucose homeostasis [24]. *A. muciniphila* is gradually being recognized as a possible therapeutic target for T2D and improving glycemic control.

A study by Zhang et al. in 2018 has shown that liver function was improved in streptozotocin-induced type 2 diabetic rats orally administered with live or pasteurized *A. muciniphila* compared to normal rats [25], and *A. muciniphila* enhanced resistance to glucose/lipotoxicity, alleviated oxidative damage, and reduced low-grade inflammation, thereby improving diabetic symptoms. Meanwhile, the administration of *A. muciniphila* supplementation to mice reduced blood serum inflammatory markers such as TNF-α, lipopolysaccharide (LPS), and plasminogen activator inhibitor-1 (PAI-1) [25]. LPS, also known as endotoxin, is a potent inducer of inflammation that induces the secretion of pro-inflammatory cytokines, leading to altered intestinal permeability, reduced insulin sensitivity, and impaired glucose tolerance [26,27]. *A. muciniphila* reduced LPS-binding protein (LBP) levels in liver and muscle, decreased the amount of phosphor-JNK, and increased IKBA protein expression, suggesting that inactivation of LPS/LBP downstream signaling leads to anti-inflammatory effects [28,29]. This is supported by increased concentrations of the anti-inflammatory factor α-tocopherol and β-sitosterol. *A. muciniphila* treatment of T2D has also been associated with TLR expression, and a recent study showed that *A. muciniphila* improved β-cell secretory function, apoptosis, and differentiation and thus repaired the intestinal barrier in HFD-induced prediabetic rats through TLR2- and TLR4-mediated signaling pathways [30].

Diabetes is characterized by elevated levels of PAI-1 [31], which is also linked to obesity and insulin resistance. Insulin resistance is one of the hallmarks of T2D, which increases PAI-1 expression. The overexpression of PAI-1 mediates the increase in blood coagulation induced by hyperglycemia. A previous systematic review and meta-analysis showed that PAI-1 levels were significantly elevated in patients with T2D compared to controls, revealing that PAI-1 levels are a risk factor for the development of T2D [32]. Zhang et al. showed that decreased PAI-1 in diabetic rats orally administered with *A. muciniphila* was accompanied by decreased TNF-α, which is similar to the results of Tang S et al. [33]. Meanwhile, it was found that diabetic rats have a higher glucagon-like peptide-1 (GLP-1) production than normal rats. And GLP-1 enhances glucose-induced insulin release, inhibits glucagon secretion, and protects β-cells from apoptosis [34]. Oral administration of *A. muciniphila* reduces excess GLP-1, highlighting the potential role of *A. muciniphila* in reducing the risk of insulin resistance.

Obesity is a major risk factor for T2D and *A. muciniphila* also plays a role in lipogenesis and gluconeogenesis. *A. muciniphila* supplementation significantly reduced the expression of genes associated with fatty acid synthesis and transport in liver and muscle [28], suggesting that *A. muciniphila* supplementation reduces lipid accumulation in liver and muscle, thereby improving insulin sensitivity. Also, endoplasmic reticulum (ER) stress in liver and muscle was alleviated by *A. muciniphila*. In addition to classical obesity-associated T2D and T1D, a proportion of T2D patients nonetheless maintain underweight or normal weight [35,36].

Compared to obese T2D patients, lean type 2 diabetic patients have different clinical characteristics and risks of diabetic complications. Macrogenomics and targeted metabolomics in lean T2D patients showed a reduced abundance of *A. muciniphila* [37]. In mouse models, supplementation with live *A. muciniphila* protected mice from glucose intolerance injury by modulating β-chenodeoxycholic acid (β-CDCA) levels, restoring insulin secretion, and enhancing fibroblast growth factor 15/19 (FGF15/19) expression, causing the subsequent stimulation of glycogen synthesis and inhibiting gluconeogenesis.

Refractory diabetes (RT2D) is also defined as T2D, and patients with RT2D are unable to maintain stable blood sugar levels. Only a tiny number of studies have focused specifically on the gut microbiota in refractory diabetes. *A. muciniphila* and *Fusobacterium* were significantly reduced in patients with RT2D [26]. Glycosylated hemoglobin A1C (HbA1c), a reliable indicator of blood sugar levels [22], and *A. muciniphila* relative abundance were significantly and negatively correlated with one another, and *A. muciniphila* reduction was positively correlated with the risk of microvascular and macrovascular complications. *A. muciniphila* may be crucial for maintaining glucose homeostasis, and the decreased abundance of *A. muciniphila* is a biomarker of glucose intolerance. *A. muciniphila* were able to modulate the negative effect of the inflammatory cytokine IFNγ on glucose tolerance, which was significantly increased in IFNγ-deficient mice, an effect that could be reversed by the reintroduction of IFNγ. Indeed, the effect of IFNγ on glucose tolerance is mediated by *A. muciniphila*, a member of the mouse gut microbiota. Mice with IFNγ knockout and without *A. muciniphila* in the flora did not show an improvement in glucose tolerance [38]. Researchers have identified the immune-related GTPase family M (Irgm1) as an IFNγ-regulated host gene responsible for controlling the level of *A. muciniphila* in the gut. Surprisingly, IFNγ was also found in human subjects, and *A. muciniphila* was associated with gene expression levels regulated by IFNγ and glucose tolerance in humans. Another study found that the pro-inflammatory cytokine IL-36 increased the secretion of colonic mucus and promoted the growth of *A. muciniphila*. Under normal chow feeding conditions, mice knocked out of IL-36Ra (IL-36 receptor antagonist) showed increased levels of IL-36 cytokine gene expression compared to wild-type mice, significantly improving hyperglycemia and insulin resistance. Interestingly, the protective effect was associated with an increase in the number of *A. muciniphila* [39].

#### 2.2.2. *A. muciniphila* Outer Membrane Protein and T2D

In addition to *A. muciniphila* itself, researchers are increasingly concentrating on the active ingredients of *A. muciniphila* that are truly beneficial and remain stable even when the bacterium itself is inactivated [7], with the hope of developing some potent medications to treat diseases. In 2017, scientists identified a specific protein present in the outer membrane of *A. muciniphila*, called Amuc_1100 [38]. Many studies have shown that Amuc_1100 can partially generalize the beneficial effects of *A. muciniphila* in diabetic mice [38,39,40]. Importantly, this protein retains the active conformation at pasteurized temperatures [39], thus explaining why pasteurized *A. muciniphila* remains effective in mouse and human experiments.

Plovier H et al. demonstrated that pasteurized *A. muciniphila* ameliorated insulin resistance and dyslipidemia, which was attributed to the interaction between Amuc_1100 and TLR2 [40]. Another study reported similar results where Amuc_1100 enhanced epithelial barrier function by activating TLR2 and TLR4 [41]. Another study also reported that Amuc_1100 could promote the expression of the 5-HT synthesis rate-limiting enzyme Tph1 in RIN-14B cells through direct interaction with TLR2, enhance 5-HT biosynthesis and extracellular availability, and restore gt microbiota diversity [42]. The regulation of 5-HT concentration in *A. muciniphila* through its outer membrane protein Amuc_1100 is one of the important pathways by which it improves or affects metabolic disorders, explaining the intrinsic mechanism of *A. muciniphila* in alleviating and treating obesity and diabetes. Recent studies also suggest that Amuc_1100 may play an important role in regulating host amino acid metabolism. In a mouse model of acute pancreatitis (AP), Amuc_1100 modulated the composition of the intestinal flora and restored tryptophan metabolism to reduce the severity of AP [43]. In addition, Amuc_1100 reduced colonic inflammation by up-regulating kynurenine (Kyn), which lowered 2-pyridinecarboxylic acid (PIC) levels and the PIC/Kyn ratio [44].

The above findings reveal that pasteurized *A. muciniphila* and outer membrane proteins still improve metabolism in obese, diabetic, acute pancreatitis, and colitis mice, showing strong effects on weight loss, fat loss, lipids, and markers of insulin resistance. *A. muciniphila* may act as an immunomodulator to reduce the risk of developing T2D.

#### 2.2.3. *A. muciniphila*-Secreted Protein and T2D

The mechanism by which *A. muciniphila* improves host obesity and glucose homeostasis is gradually being revealed. Researchers found that the administration of *A. muciniphila* to high-fat diet (HFD) mice stimulated the intestinal release of the gastrointestinal hormone GLP-1 and enhanced glucose homeostasis in mice. To further elucidate the active substance in *A. muciniphila* that promotes GLP-1 secretion, researchers treated human intestinal secretory L cells (NCI-H716) with cell-free supernatants and bacteriophages from cultured *A. muciniphila* separately and found that only the cell supernatant promoted GLP-1 secretion. The impact was determined to be unique to *A. muciniphila* since it was tested with 47 additional probiotics, including *Lactobacillus* and *Bifidobacterium*, and none of them were found to have a similar effect. After isolating various chemicals generated by *A. muciniphila*, researchers found that the P9 protein, with a molecular weight of 84 kDa, has the greatest impact on increasing GLP-1 secretion [45,46]. The P9 protein, when bound to its ligand intercellular adhesion molecule 2 (ICAM-2), improved glucose homeostasis by activating the GLP-1R signaling pathway and IL-6. These findings support our understanding of how *A. muciniphila* improves host diabetes and establish the groundwork for fresh approaches to treating disorders that are connected to it. Interestingly, the researchers found that the P9 protein is not the only mechanism for *A. muciniphila* to regulate glucose homeostasis. After giving Amuc_1100 to NCI-H716 cells, the researchers examined the expression of GLP-1 and found that Amuc_1100 also promoted the secretion of GLP-1. Notably, when the effects of the Amuc 1100 protein were compared, it was discovered that the P9 protein had a stronger effect at the same dose.

A recent study reported that a previously uncharacterized protein secreted by *A. muciniphila*, Amuc_1409 [47], improves intestinal homeostasis by interacting with E-calmodulin to promote the dissociation of the E-calmodulin/β-catenin complex, leading to the activation of Wnt/β-catenin signaling. The above findings support the notion that extracellular proteins secreted by probiotics may act as modulators of host–microbiome interactions, thereby positively impacting host health.

#### 2.2.4. *A. muciniphila*-Derived Extracellular Vesicles (AmEVs) and T2D

Increased intestinal permeability is a characteristic feature of T2D patients, which promotes the translocation of intestinal microbial metabolites into the blood and results in metabolic endotoxemia. The positive regulation of mucus thickness and intestinal barrier integrity by *A. muciniphila* may be crucial for their probiotic activity. AmEVs were found to improve glucose homeostasis [48,49], obesity [50], and postoperative cognitive dysfunction [51] in mice by improving intestinal barrier integrity. Gavage of AmEVs enhanced intestinal tight junctions and improved glucose tolerance in HFD-induced diabetic mice, suggesting that AmEV treatment can improve metabolic function in diabetic mice [48,49]. This conclusion was further supported by the study of Ashrafian F et al. The administration of AmEVs reduced plasma total cholesterol and glucose levels in HFD-induced obese mice. AmEVs replicated some of the beneficial effects of bacteria. However, one point that needs to be addressed in studies with AmEVs is how their dose correlates with the number of *A. muciniphila* cells used [3].

### 2.3. Role of A. muciniphila in GDM

Diabetes is categorized into three main subtypes: T1D, T2D, and GDM [52]. GDM is characterized by the development of glucose intolerance during pregnancy.

The current evidence suggests that GDM is associated with specific changes in the intestinal microbiota. An earlier study showed that healthy pregnant women had a higher abundance of Bacteroides, Parabacteroides, Roseburia, Dialister, and Akkermansia compared to patients with GDM [53]. Li M et al. conducted a prospective cohort study enrolling a total of 55 Chinese pregnant women to assess gut microbiota dynamics during pregnancy, and the GDM group showed increased abundance of *Ruminococcus_gnavus*, *Akkermansia_muciniphila*, *Alistipes_shahii*, *Blautia_obeum*, and *Roseburia_intestinalis* [54].

Dietary interventions have been tested in human and animal studies to increase the intestinal abundance of *A. muciniphila*. In mice with GDM, the use of prebiotic fibers such as xylo-oligosaccharide (XOS) has been associated with the growth of intestinal Akk. muciniphila, and XOS improved insulin resistance in mice with GDM by decreasing serum TNFα, IL-1β, IL-15, and LPS, and increasing *A. muciniphila* abundance [55].

## 3. Modulation of Diabetes Development by Drug-Induced Changes in the Abundance of *A. muciniphila*

According to recent research, the mechanism of action of diabetes-related therapeutic agents may be influenced by the abundance of *A. muciniphila*. Table 1 describes the contribution of different drugs to diabetes by modulating intestinal *A. muciniphila*. There is a correlation between diabetes and intestinal microorganisms such as *A. muciniphila*, which can be modified by metformin [24,25,26]. Metformin has been shown to be one of the most widely used drugs for the treatment of diabetes, especially for the treatment of patients with T2D. Numerous mechanisms, such as decreased gluconeogenesis, improved insulin sensitivity, and greater peripheral glucose absorption, have been proposed to explain the benefits of metformin [56,57]. Early research showed that metformin therapy decreased blood glucose levels in HFD mice and raised *A. muciniphila* populations. In an in vitro experiment, feces were collected from metformin-treated and control mice, and it was discovered that the proportion of *A. muciniphila* was much higher in the metformin culture group [58]. Shin et al. observed that after giving metformin to HFD mice, the animals’ blood glucose levels significantly improved and the mice had increased amounts of *A. muciniphila*. Similarly, HFD mice treated orally with *A. muciniphila* but not metformin showed improved tolerance to glucose and attenuated adipose tissue inflammation, which was dependent on the presence of Foxp3 regulatory T cells (Tregs) in visceral adipose tissue [59]. Intestinal flora of diabetic patients on metformin was examined by De La Cuesta-Zuluaga et al., who discovered that these individuals had 3.4 times more A.muciniphila in their intestines than those not receiving this treatment [60], suggesting that intestinal microorganisms like *A. muciniphila* may influence the probiotic benefit of metformin on diabetes patients. These results indicate that *A. muciniphila* may play a key role in glucose metabolism and mediate the effects of metformin, giving a new mechanism for the investigation of metformin in the treatment of patients with T2D.

Metformin, with its low cost and favorable safety profile, is the first-line treatment for T2D [61]. Recent studies have shown that metformin has a wide range of benefits besides its therapeutic effect on diabetes, including anti-intestinal damage [62], anti-colitis [63], and anti-cognitive impairment [64,65]. *A. muciniphila* protected the intestine from radiation-induced damage by secreting propionic acid, and the application of probiotic modulators, such as metformin, may be an effective therapeutic approach to protect the radiation-damaged intestine [62]. Metformin significantly increased the relative abundance of *Lactobacillus* and *Akkermansia* species and increased the expression of mucin2, a mucus barrier protein, which attenuated the symptoms of ulcerative colitis in mice [63]. Notably, an important complication of T2D is an increased risk of cognitive impairment and dementia. A study demonstrated that metformin-mediated *A. muciniphila* in the gut microbiota improved cognitive function in aged mice by reducing the pro-inflammatory cytokine IL-6 [64]. A recent clinical trial found that metformin treatment led to an increase in *A. muciniphila* and also assessed the association between the *A. muciniphila*/R. ilealis ratio and cognition, finding that the *A. muciniphila*/R. ilealis ratio was associated with improved memory scores in males but not females, and that sex hormone-induced differences in intestinal flora between men and women could explain these results [65].

Metabolic disorders such as insulin resistance and diabetes mellitus are strongly associated with obesity and non-alcoholic fatty liver disease (NAFLD). T2D and NAFLD may share a common pathophysiological basis, and both are associated with abnormalities in glucose–lipid metabolism, insulin resistance, obesity, inflammation, etc. [66]. GLP-1 receptor agonists (GLP-1RAs) are considered an option for obesity treatment and are common hypoglycemic agents [67]. A study of metformin and GLP-1RAs in patients with T2D shows that patients treated with GLP-1RAs have a higher abundance of *A. muciniphila*, and the relative abundance of *A. muciniphila* correlated significantly with the duration of the subject’s diabetes, with a higher abundance of *A. muciniphila* in short- and intermediate-term patients [68]. A later study confirmed that liraglutide treatment increased the abundance of *A. muciniphila* in an obese mouse model, reflecting the amelioration of NAFLD by GLP-1RAs [69]. Meanwhile, a clinical trial of liraglutide in the treatment of T2D combined with NAFLD found that liraglutide significantly reduced fasting glucose (FPG), 2 h glucose (2hPG), glycosylated hemoglobin (HbA1c), aspartate aminotransferase (AST)/alanine aminotransferase (ALT), and lipofuscin in patients with combined T2D and NAFLD, AST/ALT, and APN values and was superior to metformin in terms of ALT levels [70]. In another prospective, randomized, placebo-controlled study, 26 weeks of treatment with liraglutide plus metformin (2000 g/day) was more effective than placebo in reducing IHCL, SAT, and VAT in patients with T2D and NAFLD [71]. In addition, another clinical study demonstrated that GLP-1RAs can be used for the full treatment of T2D when patients fail to meet their metformin treatment goals [72].

In addition, certain antibiotics can also affect the windiness of *A. muciniphila* in the intestine. Andrographolide, a natural antibiotic drug, can alter the composition of the intestinal microbial community, as evidenced by an increase in the abundance of *A. muciniphila* and an increase in the ratio of *Bacteroidetes*/*Firmicutes*. Andrographolide has several beneficial effects on diabetes and its complications, reducing metabolic endotoxemia, enhancing intestinal barrier function and increasing the microbial species of *A. muciniphila* prevent hyperglycemia [73].

Clinically, Chinese herbal decoctions are also frequently utilized in the treatment of patients with T2D. Rhubarb extracts (rich in polyphenols) [74] prevent diet-induced obesity, glucose intolerance, and adipose tissue inflammation, and maintain intestinal barrier integrity, the effects of which are closely related to the crosstalk between *A. muciniphila* and Reg3γ in the colon. In addition, Huang-Lian-Jie-Du-Decoction [75], JinQi Jiangtang Tablets [76], etc., can improve insulin resistance in T2D patients as well as glucose homeostasis, reduce inflammatory stress, and raise the abundance of *A. muciniphila* in the colon. *A. muciniphila* may be one of the mechanisms of Chinese herbal decoction for the treatment of T2D.

As an emerging probiotic, the application of *A. muciniphila* in the food industry has been the subject of considerable debate. Currently, *A. muciniphila* WB-STR-0001 has been successfully applied as a probiotic medical food to improve the symptoms of T2D [77,78]. A novel probiotic preparation, WBF-011 [79], which contains intestinal bacteria deficient in T2D patients, including *A. muciniphila* WB-STR-0001, *Bifidobacterium infantis,* and butyric acid-producing *Anaerobutyricum hallii*, *Clostridium beijerinckii*, *Clostridium butyricum*, and prebiotic dietary fiber inulin. After 12-week administration of the novel prebiotic formulation to participants with T2D using metformin, patients in the WBF-011 group showed significant improvements in the primary endpoint (curve (AUC)) and secondary endpoints (glycated hemoglobin, glucose incremental AUC) compared to the placebo group, and no major safety or tolerability issues were observed. This is the first randomized controlled trial of five strains administered to human participants with T2D, and WBF-011 is the first probiotic special medical food with the addition of *A. muciniphila*. The increase in butyrate producers and *A. muciniphila* abundance induced by metformin treatment is thought to be a supportive mechanism for the efficacy of this important anti-diabetic drug [80]. Whether this beneficial effect is mediated by *A. muciniphila* alone needs to be investigated, as other bacteria, including *Anaerobutyricum hallii* and inulin, were also taken concomitantly. To investigate the effects of single versus multiple probiotic strains on glycemic control, assess their value as adjuncts to hypoglycemic medications, and clarify how these effects may be mediated by changes in the ecology of a specific gut microbiota, large, long-term randomized controlled trials will need to be conducted. In addition, the study was conducted in patients previously taking metformin, and the results suggest that WBF-011 may be more beneficial than metformin, which failed to successfully regulate these patients’ conditions.

**Table 1:** Mechanisms of the effect of different drugs on diabetes mellitus by modulating the intestinal *A. muciniphila*. GLP-1RAs: GLP-1 receptor agonists.

Drugs	Models	Periods	Effect of Treatment on Microbes	Beneficial Changes	References
Metformin	HFD mice	10 weeks	*A. muciniphila* ↑ *Clostridium cocleatum* ↑	Improved serum glucose levels	[58]
Metformin	Normal chow diet or HFD mice	6 weeks	*A. muciniphila* ↑	Reduced serum LPS, enhanced glucose tolerance, attenuated adipose tissue inflammation	[59]
Metformin	28 patients with T2D	Not mentioned	*A. muciniphila* ↑ *Butyrivibrio* ↑ *Bifidobacterium bifidum* ↑ *Megasphaera* ↑	Significant differences were found in the comparison of β-diversity of microbial groups	[60]
GLP-1 AR (i.e., liraglutide)	37 patients with T2D (18 treated with metformin and 19 treated with GLP-1 mimetics)	6 weeks	*A. muciniphila* ↑	Patients receiving a GLP-1 agonist had higher Akkermansia abundances than those on metformin	[68]
Andrographolide	db/db mice	8 weeks	*A. muciniphila* ↑ *Bacteroidetes*/*Firmicutes* ↑	Improved glucose tolerance and insulin resistance, and reduced redox disorders and inflammation	[73]
Rhubarb extract	HFHS mice	8 weeks	*A. muciniphila* ↑	Increased Reg3γ expression in the colon, prevented insulin resistance and liver steatosis	[74]
Huang-Lian-Jie-Du-Decoction	HFD- and streptozotocin-induced type 2 diabetic rats	4 weeks	*A. muciniphila* ↑ *Parabacteroides* ↑ *Blautia* ↑ *Aerococcus* ↓ *Staphylococcus* ↓ *Corynebacterium* ↓	Improved impaired glucose tolerance	[75]
JinQi Jiangtang Tablet	Streptozotocin-induced type 2 diabetic rats	5 weeks	*A. muciniphila* ↑ *Desulfovibrio* ↓	Down-regulated fasting glucose and HbA1c levels, reduced TNF-α and IL-6 levels, increased insulin sensitivity, and inhibited inflammation	[76]
WBF-011	76 patients with T2D	Average of 78 days	Not mentioned	Improved glycated hemoglobin and blood glucose levels	[79]

↑ shows increase in microbiota, ↓ shows decrease in microbiota.

## 4. Modulation of Diabetes Development by Diet-Induced Changes in the Abundance of *A. muciniphila*

Diabetes is a multifactorial disease involving genetic and environmental factors. In addition to gut flora, medication use, and genetic and environmental factors, one opportunity for the future is to offer personalized diets to regulate the health of patients with diabetes [81,82,83,84,85]. Furthermore, it has been demonstrated that the diet can directly affect the gut microbiota by altering its metabolic activity or composition to slow the onset of disease or promote homeostasis. *A. muciniphila*, a Gram-negative bacterium that utilizes mucin as its sole source of carbon, nitrogen, and energy, has been found to modulate the abundance of *A. muciniphila* by dietary nutrients and provide a microenvironment for their growth and reproduction [86]. Targeted dietary intervention increasing the number of *A. muciniphila* in the intestinal tract, hence exerting their probiotic properties, are also the focus of current research applications [87] (Table 2).

Dietary polyphenols are natural antioxidants and both the antioxidant and antimicrobial activities of dietary polyphenols may potentially reshape the ecology of the gut microbiota. Many clinical studies have shown that polyphenols or polyphenol-rich foods, such as cranberry and grape polyphenols, significantly alleviate metabolic syndrome and improve glucose tolerance [88,89,90,91]. One of the extra frequently discussed methods of boosting *A. muciniphila* abundance is cranberry extract (CE). In mice fed a high-fat–high-sucrose diet (HFHS, lacking soluble fiber), Anhê et al. found that CE attenuated metabolism, improved insulin sensitivity, and reduced glucose-induced hyperinsulinemia, thereby improving insulin sensitivity, which was associated with an increase in the abundance of *A. muciniphila* [92]. Another study showed that CE also alleviated insulin resistance and hepatic steatosis, findings that were associated with improved intestinal–liver homeostasis and multiplied the abundance of *A. muciniphila* in the intestine [88], with the intestinal–liver axis being the main target of cranberry extract. A trial by Roopchand DE et al. [89] confirmed that HFD-fed C57BL/6J mice supplemented with 1% grape polyphenols (GPs) promoted the growth of *A. muciniphila*. GPs alter the composition of the intestinal microbiota, thereby attenuating glucose tolerance and intestinal and systemic inflammation. Another subsequent study showed that mice fed GP extract (GPE) and grape proanthocyanidins showed increased *A. muciniphila* in the feces and cecum mucus, independent of specific intestinal gene expression changes, but at a rate dependent on the basal abundance of *A. muciniphila* [90]. In addition, chlorogenic acid (CGA) has also been shown to be associated with *A. muciniphila* regulation, with CGA promoting *A. muciniphila* growth, inhibiting body weight gain, and significantly reducing adipose tissue abnormalities in mice [93]. There are also some inconsistencies in the literature, such as dietary flaxseed, which has been shown to lead to a reduction in *A. muciniphila* abundance [94,95]. Such contradictory results highlight the heterogeneity of polyphenol compositions. However, it is clear that fiber supplementation may be beneficial in the treatment of diabetes. Taken together, these results suggest that dietary polyphenols have prebiotic properties. Nevertheless, future investigations are essential to comprehensively elucidate the underlying mechanisms and to optimize the utilization of polyphenols for the enhancement of gut health and the prevention of diseases.

Dietary fiber has been reported to play an important role in the regulation of the intestinal microbiota. Modulation of the host microbiota through long-term adherence to a high-fiber diet could be a potential therapeutic approach to improve T2D symptoms. Several animal studies have shown that the oral administration of oligofructose, a prebiotic, promoted the growth of *A. muciniphila* in mouse models [24,96]. Oral prebiotic supplementation increased the abundance of *A. muciniphila* in ob/ob mice by more than 80-fold, and prebiotic treatment improved glucose tolerance and insulin sensitivity [96]. Another study found that a high-fat diet in DIO mice resulted in a 100-fold reduction in *A. muciniphila* [24], and that oligofructose supplementation restored their concentrations and improved glucose homeostasis and reversed metabolic endotoxemia and related diseases. Mannans can increase the hypoglycemic effect of metformin, and metformin and mannans have a synergistic effect on improving insulin resistance and glucose tolerance, which increased the abundance of *A. muciniphila* [97]. Carboxymethylated wheat bran dietary fiber (DF) has also been shown to improve hypolipidemia and hypoglycemic effects. Modified DFs fed to T2D mice improved insulin receptor activity and insulin signaling transduction to inhibit hepatic gluconeogenesis, while promoting the synthesis of SCFAs, driving gastrointestinal hormone and insulin regulation of blood glucose, and reducing blood glucose levels in diabetic mice. 16S rDNA sequencing showed that the abundance of *A. muciniphila* was significantly increased in mice fed with modified DFs, and that DFs improved gut microbiota diversity in diabetic mice [98]. Consistent with animal studies, an association between the abundance of this bacterium and dietary fiber has been reported in human studies. Sodium butyrate and inulin supplementation significantly increased the abundance of *A. muciniphila* in diabetic patients, and dietary supplementation significantly decreased TNF-α mRNA expression, as well as high-sensitivity C-reactive protein and malondialdehyde, both markers of systemic inflammation and oxidative stress [99].

Despite the fact that the beneficial effects of *A. muciniphila* have been widely recognized in metabolic diseases, conflicting results have been found [100]. One study showed that walnut green husk polysaccharide extracts inhibited insulin and glucose levels preventing chronic high-fructose diet-induced abnormal weight gain and glucose intolerance in mice, but *A. muciniphila* abundance was reduced [101]. Another study on colitis showed that β-glycosidic polysaccharides from pleurotus eryngii had an inhibitory effect on dextran sodium sulfate-induced colitis in mice, but the abundance of *A. muciniphila* was reduced [102]. The reasons for the various results of dietary fiber modulating the levels of *A. muciniphila* are complex, with the differences between studies attributable to the type of fiber supplement, strain specificity, and animal model differences, among other things. Despite the existence of conflicting results, it is evident that fiber supplements may have a beneficial effect on the treatment of diabetes. Future research needs to benefit human health with synbiotic products, elucidate the association between dietary fiber and *A. muciniphila*, and dissect methods of increasing fiber intake to enhance nutrient utilization.

A clinical study by Vitale M et al. [103] also found that a Mediterranean diet (rich in fiber) improved glucose metabolism and insulin sensitivity. Increased postprandial plasma butyrate concentrations were also reported, and butyrate concentrations were directly correlated with postprandial insulin sensitivity. These metabolic changes were combined with an increase in the abundance of *Intestinimonas butyriciproducens* and *A. muciniphila*. This study highlights the possible involvement of gut microbiota metabolites—such as butyric acid—and of dietary fiber as a precursor in improving glucose metabolism and insulin sensitivity. Another randomized controlled trial found that dietary intervention with functional foods (rich in polyphenols, plant proteins, fiber, etc.) greatly improved the fecal microbiota of patients with T2D, with increased abundance of *Faecalibacterium prausnitzii* and *A. muciniphila*, both of which have anti-inflammatory properties [104]. Significant reductions in glucose, triglycerides, total cholesterol, and HbA1c were observed after treatment with the functional food combinations, reducing metabolic endotoxemia. Dietary interventions with functional foods offer potential therapeutic approaches for dyslipidemia and glucose homeostasis. In conclusion, appropriate dietary interventions may affect *A. muciniphila* abundance, highlighting a new potential mechanism by which understanding the link between different types of dietary interventions and changes in *A. muciniphila* abundance could help develop future nutritional recommendations and design medical nutrition therapies to help prevent and treat *A. muciniphila*-related diseases, such as diabetes and obesity.

**Table 2:** Effect of different dietary compositions on diabetes via modulation of intestinal *A. muciniphila*. CE: Cranberry extract; GPs: grape polyphenols; DF: carboxymethylated wheat bran dietary fibers; NaBut: sodium butyrate.

Source	Models	Treatment	Effect of Treatment on Microbes	Beneficial Changes	References
CE	HFHS mice	200 mg/kg for 8 weeks	*A. muciniphila* ↑ (2% to over 30% in feces)	Improved insulin tolerance, lower homeostasis model assessment of insulin resistance, and decreased glucose-induced hyperinsulinemia	[92]
CE	DIO mice	CE (200 mg/kg, Chow + CE, HFHS + CE) or vehicle (Chow, HFHS) for 8 weeks	*A. muciniphila* ↑	Reverse HFHS diet-induced insulin resistance and hepatic steatosis	[88]
GP	C57BL/6J mice	HFD containing 1% Concord grape polyphenols	*A. muciniphila* ↑ *Firmicutes* to *Bacteroidetes* ↓	Lowered intestinal expression of inflammatory markers (TNFα, IL-6, inducible nitric oxide synthase); attenuated glucose intolerance	[89]
GPs(proanthocyanidi)	C57BL/6J mice	10 days with GPE (delivering 360 mg total PACs/kg), PAC standard (360 mg/kg)	*A. muciniphila* ↑	Attenuated glucose intolerance	[90]
Oligofructose	DIO mice	0.3 g/d with a standard diet for 8 weeks	*A. muciniphila* ↑ (100-fold decrease in feces)	Reversed metabolic endotoxemia, improved mucosal barrier function	[24]
Oligofructose	ob/ob mice	0.3 g/d with a standard diet for 8 weeks	*A. muciniphila* ↑ (80-fold decrease in feces)	Reduced plasma LPS, improved gut barrier function, improved glucose tolerance	[96]
Mannan and metformin	HFD- and streptozotocin-induced type 2 diabetic rats	5 weeks	*A. muciniphila* ↑ *Bifidobacterium pseudolongum* ↑	Improved insulin resistance and glucose tolerance	[97]
DF	HFD and streptozotocin-induced type 2 diabetic rats	10% or 2,5% modified DF into HFD for 4 weeks	*A. muciniphila* ↑ *Firmicutes* to *Bacteroidetes* ↓	Increased the serum insulin content, recovery effect on islet β-cells	[98]
NaBut and inulin	60 overweight and obese diabetic patients	600 mg/d NaBut (group A), 10 g/d inulin powder (group B), both inulin and NaBut (group C)	*A. muciniphila* ↑	Reduced TNF-α mRNA expression	[99]
Walnut green husk polysaccharide extracts	High-fructose diet-induced obese mice	200, 400, and 800 mg per kg bw (0.4 mL, i.g.) once daily	*A. muciniphila* ↓ Lachnoclostridium↓	Suppressed weight gain, liver and fat weight, TG levels, TC levels, insulin levels, and glucose levels	[101]
Mediterranean diet	Twenty-nine overweight/obese individuals of both genders, aged 20–60 years	Mediterranean diet for 8 weeks	*A. muciniphila* ↑ *Intestinimonas butyriciproducens*↑	Reduced glucose and insulin responses, improved insulin sensitivity	[103]
Functional food-based diet	81 patients with T2D	Dietary portfolio or portfolio treatment combined with reduced energy diet for 1 month	*P. Copri* ↓ *A. muciniphila* ↑ *Faecalibacterium prausnitzii* ↑	Reduced metabolic endotoxemia mediated by LPS, increased serum antioxidant activity	[104]

↑ shows increase in microbiota, ↓ shows decrease in microbiota.

## 5. Limitations of *A. muciniphila*-Related Studies

Probiotics are one of the important and effective interventions, and *A. muciniphila* intervention may help us to prevent diabetes to some extent, but it is far from enough. First, the exploration of *A. muciniphila* provides a new perspective to reveal and analyze the mechanism of diabetic disease, and the research results achieved so far also provide more possibilities to improve human health. However, due to the complexity of *A. muciniphila*, the mechanisms of action involved in the development of diabetes are not fully understood and there are still many limitations, unknowns, controversies, and challenges.

Several studies have shown that *A. muciniphila* is not beneficial in all cases. There are some top journals that have similarly reported the association of a high abundance of *A. muciniphila* with disease, and the contradictory findings show that there is still a lot of unanswered work on *A. muciniphila*. Chassaing et al. observed that *A. muciniphila* increased intestinal permeability in IL10^−/−^ mice, leading to impaired mucus-protective function and bacterial adhesion and increased inflammatory microbiota [105]. Seregin et al. found that repeated gavage of *A. muciniphila* led to an increase in the severity of colitis in SPF IL10^−/−^ mice [106]. As an intestinal commensal bacterium, *A. muciniphila* is not pathogenic per se, but whether it acts synergistically with other bacteria to lead to disease occurrence, and how to safeguard it from toxic effects in clinical applications, are concerns.

It is important to understand the causal relationship between *A. muciniphila* and diabetes, and further unified studies are needed. Previous interventions of *A. muciniphila* in diabetes are usually based on animal models, and human clinical validation, feasibility and safety assessments, and individualized dosing are the key focuses that need to be studied in depth. In July 2019, the results of the first human trial of *A. muciniphila* probiotic supplementation were published in *Nature Medicine*, where a research team from Belgium conducted a 3-month trial on 32 volunteers were orally administered 10^10^ live *A. muciniphila*, pasteurized *A. muciniphila*, and placebo daily for 3 months, and the results showed that *A. muciniphila* could enhance intestinal barrier function and reduce plasma LPS [107]. This trial confirmed the safety of *A. muciniphila* and that daily oral administration of a 10^10^ cell dose of live *A. muciniphila* was safe for volunteers. A probiotic special medical food supplemented with *A. muciniphila* was first administered to T2D patients in 2020, and no significant safety or tolerability issues were observed [79]. In the past two years, researchers have conducted 90-day genotoxicity assay studies on live and pasteurized *A. muciniphila* [108,109]. Druart C et al. evaluated the safety of pasteurized *A. muciniphila*, and a level of adverse effects was not observed in a study of administration of 9.6 × 10^10^ *A. muciniphila* cells/kg weight/day) for 90 days [108]. In a recent study, Yu E et al. found no genotoxicity in a powder formulation of live, viable *A. muciniphila* assessed in an in vivo mammalian cell micronucleus assay when administered at up to 1.64 × 10^11^ CFU/kg body weight/day [109]. *A. muciniphila* can be administered directly, and these studies varied in dosage, duration, and other factors, which may affect the results, but the results of these studies provide some support that *A. muciniphila* administration is safe.

The administration of *A. muciniphila* has beneficial effects, and these effects are not reduced but enhanced after pasteurization [108]. A randomized, double-blind, placebo-controlled proof-of-concept study in overweight/obese insulin-resistant human volunteers showed that the daily oral administration of pasteurized *A. muciniphila* significantly improved several metabolic parameters [107], such as insulin levels, insulinemia, and plasma total cholesterol. Meanwhile, Ashrafian F et al. found that the biochemical parameters and biomarkers of inflammation in plasma of normal-diet mice given live and pasteurized *A. muciniphila* showed that pasteurized *A. muciniphila* had a more significant effect [110]. In contrast, Du Y et al. administered live or pasteurized *A. muciniphila* (5 × 10^9^ CFU/200 μL) to db/db mice and found that live *A. muciniphila* possessed higher efficacy in improving diabetic cognitive impairment compared to live *A. muciniphila* [111]. More extensive animal studies and human proof-of-concept studies are needed to determine what is more effective: pasteurized *A. muciniphila* or live bacteria.

## 6. Conclusions and Future Perspectives

*A. muciniphila* has been widely recognized for its relevance to host health and metabolic disorders, and its role in regulating diabetes, especially T2D, has become a hot research topic in the field of probiotics in recent years. As a next-generation probiotic, *A. muciniphila* has great potential to become a potential target or tool for early diagnosis or treatment of diabetes-related diseases. Several animal trials have shown that the administration of *A. muciniphila* may improve insulin sensitivity and glucose homeostasis in diabetic mice by reducing the incidence of insulinemia, enhancing intestinal barrier function, and promoting GLP-1 secretion. In addition to live *A. muciniphila*, similar or even stronger beneficial effects were observed in pasteurized *A. muciniphila* and its components, including Amuc_1100, Amuc_1409, AmEVs, and the secreted protein P9.

The regulation of *A. muciniphila* abundance can be accomplished in several ways, such as direct administration as a probiotic, administration of prebiotics, and interventions such as drugs and diet [112,113,114].

The researchers propose the analysis of *A. muciniphila* abundance as a tool to identify individuals who are expected to benefit from dietary and pharmacological interventions. A promising future approach could use a synergistic approach involving drugs, diet, and microbiota in the prevention and treatment of diabetes. Drugs such as metformin can stimulate the growth of the *A. muciniphila* and are therefore considered a major confounding factor in studies targeting diabetes [3]. However, considerations such as the mechanisms associated with *A. muciniphila* and the minimum required to produce beneficial effects need to be supported by additional data. This knowledge gap should be further explored in future studies. Also, several studies have found that probiotics have been proposed to confer more lasting benefits compared to the sole use of *A. muciniphila* [115]. Prebiotics and dietary fiber added to foods can be synergistic with *A. muciniphila* to help improve intestinal function in people with diabetes and others. The use of prebiotics/probiotics has been demonstrated through their reliable effectiveness evaluations, but it should be noted that not all products tested have been validated, and a number of contradictory conclusions have emerged [105,106]. More appropriate animal and human studies may reveal new insights. To explain the effectiveness of prebiotics/probiotics in the prevention and management of diseases such as diabetes, scientific and long-term randomized, placebo-controlled trials should be conducted.

## Data Availability

No new data were created or analyzed in this study. Data sharing is not applicable to this article.
